# Supplementary material for: Synchronization‐Dissipation in the Cardiorespiratory System
Source: Adv Sci (Weinh). 2026 Apr 9:e75202. Online ahead of print. doi: 10.1002/advs.75202 (PMC13335046; doi:10.1002/advs.75202)
Supplement: Supplementary file 1 — Supporting File: advs75202‐sup‐0001‐SuppMat.pdf. [file ADVS-9999-e75202-s001.pdf]

Synchronization-dissipation in the cardiorespiratory system

Josh Border, Alain Nogaret, Andrew Lefevre, Vishal Jain

Department of Physics, University of Bath, Bath BA2 7AY, UK

1. Derivation of viscoelastic and Poiseuille power densities

1.1. Viscoelastic power dissipation in capillary walls

The stress-strain relation along the capillary axis is modelled with a spring and dashpot in parallel (Voigt-Kelvin model). The stress is given by:

$$\sigma = E\epsilon + \eta\dot{\epsilon} \tag{1}$$

The first elastic term gives no energy absorption over a cycle. The second term is the only one leading to dissipation. The viscous power dissipated by a capillary of length *l* and sectional area *A* at rest is:

$$P_v = \frac{dW}{dt} = \frac{F.dl}{dt} = \frac{Fld\epsilon}{dt} \tag{2}$$

Using $F = \sigma A$, and retaining the first order terms in ϵ, one obtains:

$$P_v = \sigma_v Al.\dot{\epsilon} = \eta\dot{\epsilon}Al\dot{\epsilon} \tag{3}$$

Dividing P_v by the volume of the capillary, Al, we obtain the viscous power dissipated per unit volume of the capillary as:

$$p_v = \eta\dot{\epsilon}^2 \tag{4}$$

where η is the viscosity of the capillary wall. The viscoelastic power dissipated is thus the variance of the rate of change of axial strain on the capillary, within a constant factor. This dissipation mechanism only affects the *oscillatory component* of blood flow.

1.2. Poiseuille dissipation to laminar flow in the steady state

This is the power loss incurred through the shear stress of *laminar blood flow* on the capillary wall. Assuming μ to be the dynamic viscosity of blood; v the mean blood velocity; and l and A the length and cross-sectional area of a capillary segment at rest, Poiseuille power dissipation per unit volume of the capillary is:

$$p_v^b = 8\pi\mu v^2(1+\epsilon)/A \tag{5}$$

The ϵ term denotes the reduction in membrane area under stretch as surface tension on the capillary walls keeps volume constant. Over a strain cycle there will be no net power dissipated. Hence Poiseuille dissipation does not contribute to power loss by the oscillatory component of blood flow. It only contributes to dissipation of the static flow through the first term $8\pi\mu v^2/A$.

1.3. Numerical power estimates

Blood viscosity: $\mu = 0.003$ kg.m^{-1}.s^{-1}

Capillary wall viscosity: $\eta = 20 - 30$ kg.m^{-1}.s^{-1}

Mean blood velocity in alveoli capillary[1]: $v = 0.2 - 1.0$ mm.s^{-1}

Mean capillary radius: $r = 3.5 \times 10^{-6}$m

During systole one may assume the strain rate peaks at $\dot{\epsilon} \approx 5.0$ s^{-1} giving:

$$p_v \approx 25 \times 5^2 = 625\text{W.m}^{-3}$$

Mean Poiseuille dissipation is:

$$p_v^b \approx \frac{8\pi \times 0.003 \times (0.5 \times 10^{-3})^2}{\pi(3.5 \times 10^{-6})^2} = 490\text{W.m}^{-3}$$

Hence the power lost to viscoelastic dissipation in capillary walls is of same order as the power dissipated by laminar blood flow. The highest level of viscoelastic dissipation occurs during systole which covers ≈30% of the R-R interval.

2. Waveforms of cardiac and respiratory strain

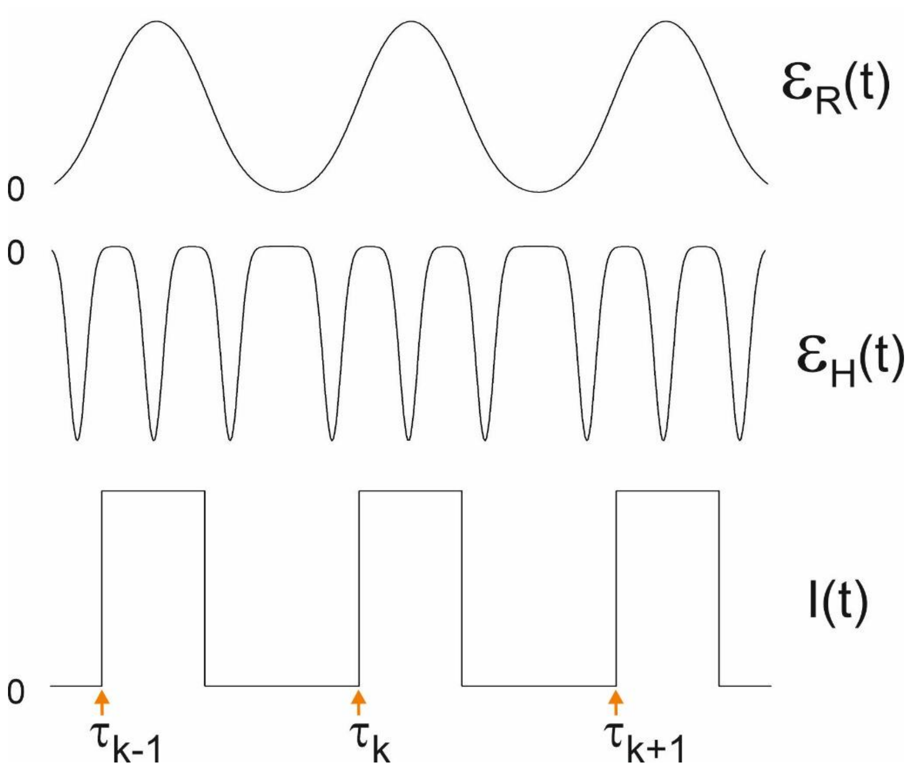

Figure S1: Indicative waveforms of the respiratory strain $\epsilon_R(t)$, cardiac strain $\epsilon_H(t)$, and lung inflation drive I(t) in the 3:1 synchronization mode. Parameters are $rsa = 25\%$, $\beta = 0.4$, $\delta = 0.5$s (1/6th of the respiration period), $\Gamma = 0.15$s (half the duration of systole ≈ 0.3s). Strain amplitudes are $\epsilon_R^0 = \epsilon_H^0 = 0.25$.

[1] Horimoto et al., *Respiration Physiology* **37**, 45-59 (1979)

3. Multimodal synchronization of the neuronal oscillator driven by breathing

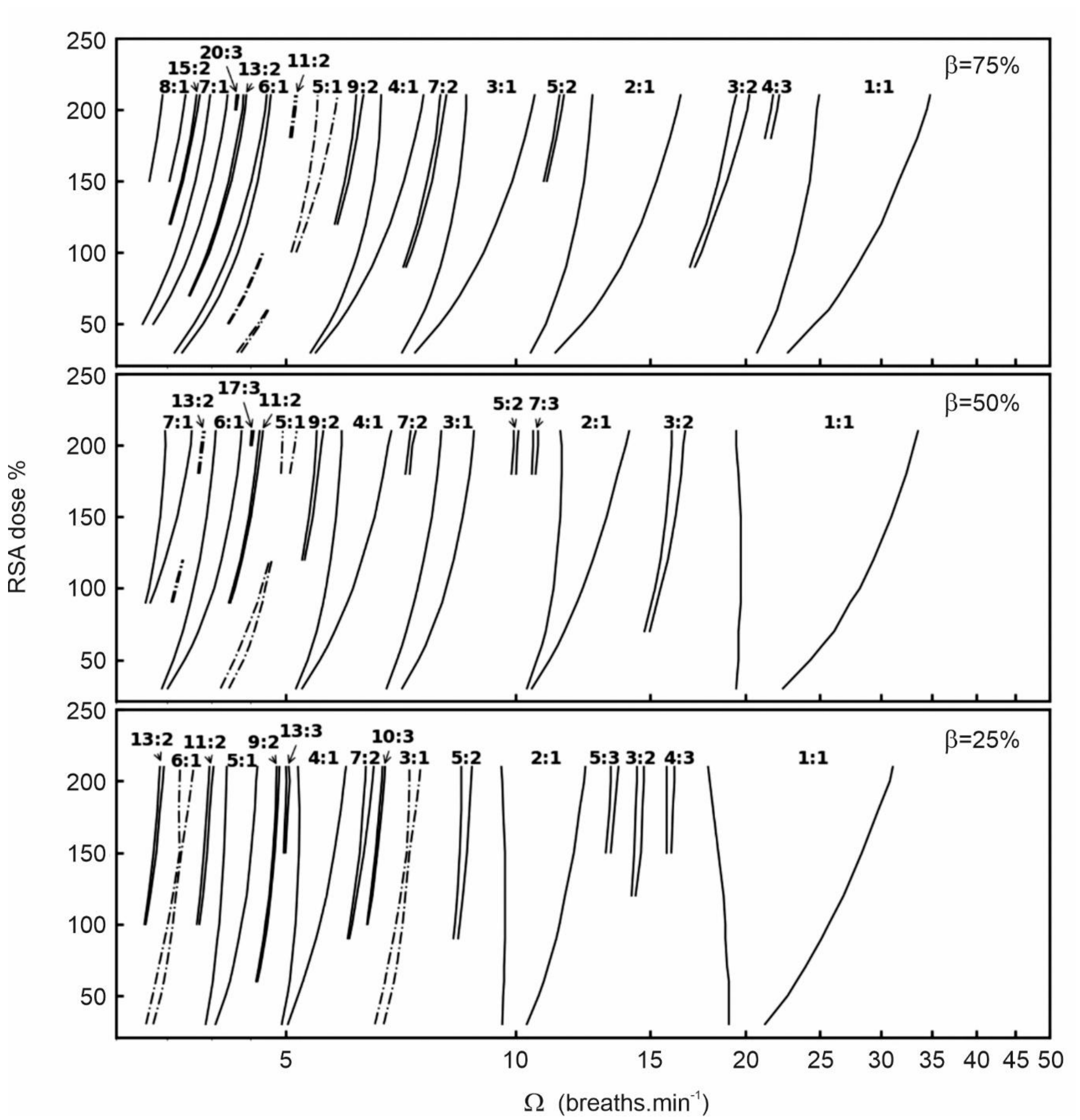

Figure S2: Multimodal synchronization of the cardiac rhythm to the breathing rhythm as a function of respiratory frequency (Ω) as the RSA dose increases (*rsa*). The *m:n* ratio decreases with increasing respiration frequency as fewer R-R intervals fit within the respiration period. The three panels from top to bottom show the effect of reducing the duty cycle of the inspiratory phase from β=75% to β=25%. Arnold tongues shift to lower breathing frequency as reducing β reduces the average cardiac frequency. The same is true when the RSA dose decreases.

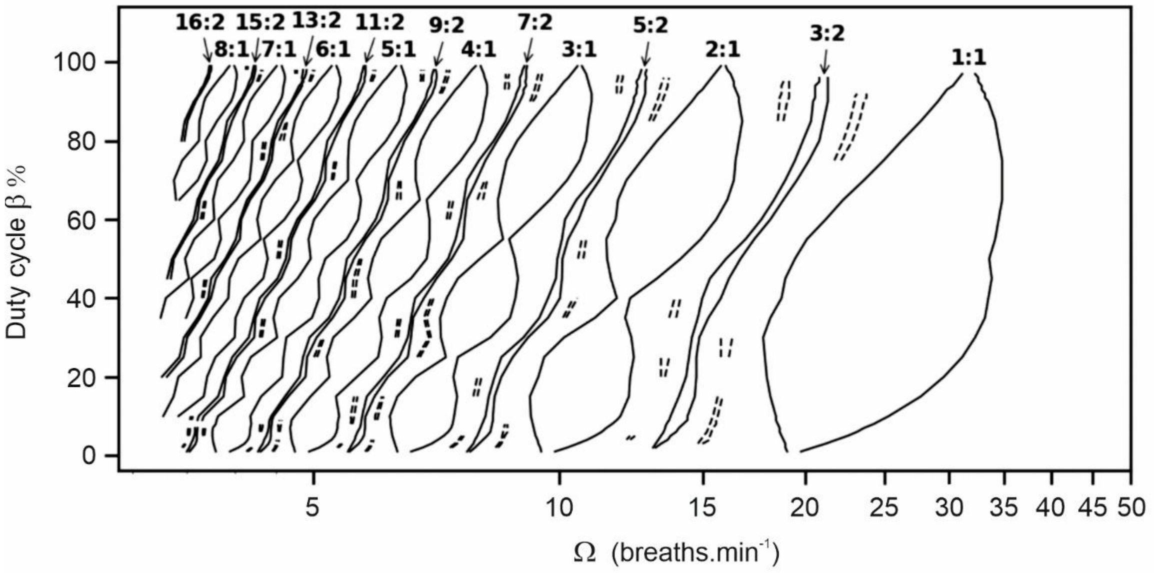

Figure S3: Multimodal synchronization of the cardiac rhythm to the breathing rhythm. The RSA dose was *rsa* = 150%. Arnold tongues happen to shift towards higher respiration frequency as the duty cycle increases. This is because respiration frequency locks to the mean cardiac frequency ω that increases with increased average stimulation $\langle I(t) \rangle$ over a cycle. The heart-respiration coupling is maximum at β≈50% where the 1:1 Arnold tongue is widest.

4. Multimodal synchronization under aperiodic breathing

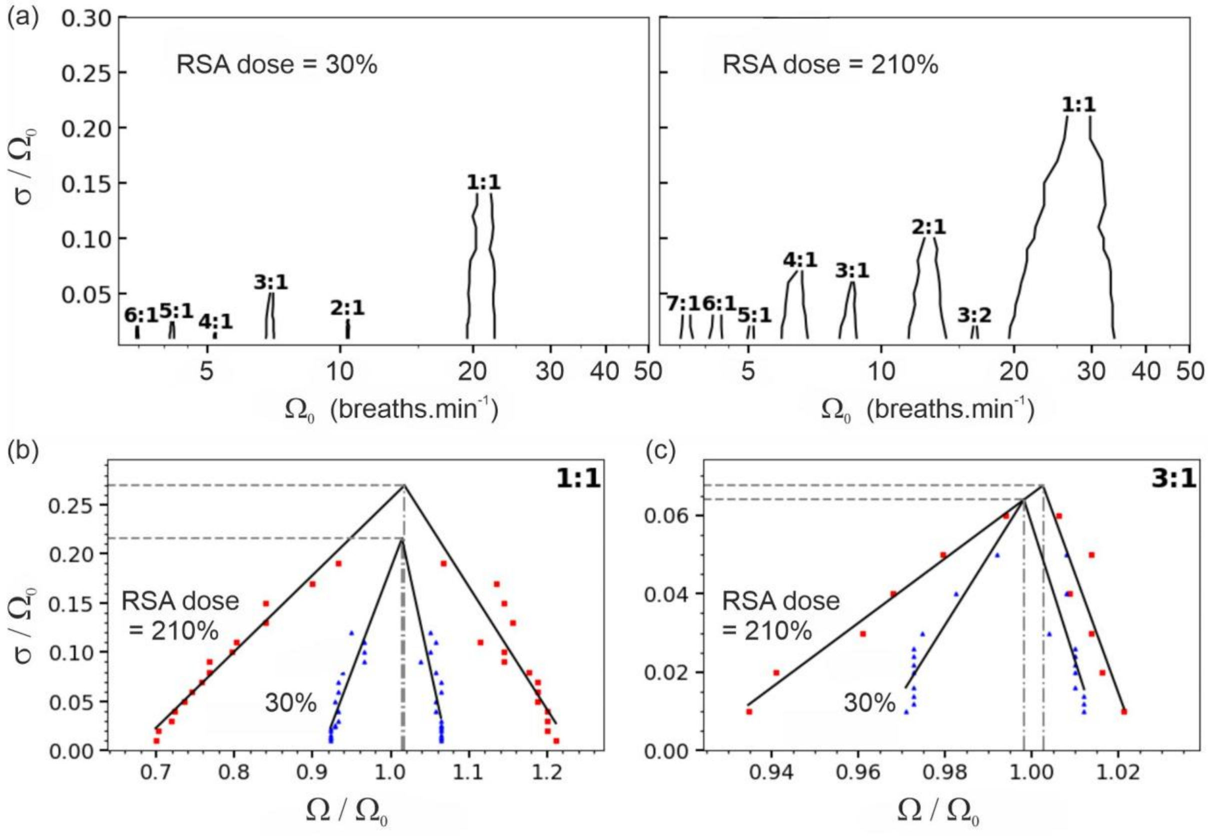

Figure S4: (a) Dependence of the width of *m*:*n* synchronization regions on the standard deviation (σ) of breathing intervals. Increasing the standard deviation of respiratory intervals reduces the width of synchronization regions. The greater the RSA dose, the more robust cardiorespiratory synchronization is to variability in breathing intervals. (b) Arnold tongue 1:1 calculated at RSA dose rsa = 30% and 210%. (c) Same for Arnold tongue 3:1. Ω_0 is the mean breathing frequency. Ω-Ω_0 is the frequency detuning from mean.
